# Determine the relationship between perceived social support and depression level of patients with diabetic foot

**DOI:** 10.1186/s40200-015-0168-8

**Published:** 2015-07-16

**Authors:** Ebru Yildiz, Türkinaz Aşti

**Affiliations:** Nursing Department, Batman University School of Health, Batman, Turkey; Nursing Department, Bezmi Alem University Faculty of Health Sciences, İstanbul, Turkey

**Keywords:** Diabetes, Diabetic foot, Depression, Social support

## Abstract

**Background:**

As a lifelong disease, diabetes impairs the quality of life by limiting the eating and drinking habits and by bringing out the risk of kidney, eye, cardiovascular and diabetic neurological diseases in the long run. Loss of health might result in mourning, grief, rebellion, denial, anxiety, rage and sometimes these feelings might overcome the patient’s coping skills leading to depression [Clinical Psychiatry 11 (Suppl 3) 3-18, 2008]. How individuals suffering from depression perceive and interpret the incidents around them is also important [Rel. Scie. Acad. J. III, 2: 129-152, 2003]. Accordingly, the determination of the correlation of the depression with the perceived social support level by the patients with diabetic foot was programmed and performed in order to take essential precautions, to generate proper solutions and treatment process and to make supportive plans for patients with developing diabetic foot and depression.

**Methods:**

The data was obtained from 128 patients who applied to hospital within the scope of research between July 1st 2011 and January 31st 2012 that were diagnosed with diabetes and had diabetic foot. Pearson chi-square, Fisher Exact and Likelihood ratio, chi-square, Student *t* test and one way analysis of variance, Levene’ s test, One way ANOVA, Welch and Games Howell tests were used in the analysis and evaluation. The data was collected by meeting face to face the individuals and by making use of the patient files and using the “Personal Information Form” which includes introductory information about individuals with diabetic foot, “Beck Depression Scale” which is applied to determine emotion status of individuals and “Multidimensional Scale of Perceived Social Support” which is applied to determine the level of social support individuals perceive.

**Results:**

In the performed statistical evaluation, mean scores of Beck Depression scale and MSPSS family support, friend support, special person support sub-dimension and scale total scores were found to be in negative statistical correlation (*p* < 0.01).

**Conclusions:**

In the treatment and care of the patients with diabetic foot; anxiety and depression status of the patients, as well as physical status, should also be evaluated routinely. The individuals provided to take professional care.

## Introduction

As a lifelong disease, diabetes impairs the quality of life by limiting the eating and drinking habits and by bringing out the risk of kidney, eye, cardiovascular and diabetic neurological diseases in the long run. Loss of health might result in mourning, grief, rebellion, denial, anxiety, rage and sometimes these feelings might overcome the patient’s coping skills leading to depression. Tension, anxiety and depression adversely affect the progress of the disease both via neuroendocrine mechanisms and by making it more difficult to adapt to the nutrition and exercise programs which are requirements of the treatment [1].

The risk of depression increases with severity of the disease and the additional problems caused by the disease; and the feeling of healthiness and strength might become disrupted. The emotions and behaviors of the patient suffering from depression swings between future anxiety, loss of potency, concerns about sexuality, productivity and appearance together with excessively pushing and risky behaviors. State of mind and diabetes are in a bidirectional interaction. Mood swings affect blood sugar and changes in blood sugar affect behaviors and emotions [1].

Prevalence of depression is higher in patients who develop diabetes related complication. The quality of life of the patient decreases, limitations in life and therefore dependency to others increase as complications of diabetes develop, which in turn results in the increase of severity of depression and the risk of recurrence [2]. The organs that are most adversely affected by the nerve injury and occlusive vascular disease which develop as complications of diabetes are lower extremities. Diabetic foot syndrome develops as a result of neuropathy and vascular insufficiency together and it is one of the most serious and severe complications of Diabetes mellitus (DM). For this reason, diabetic patients should be well observed for their feet, be given the necessary education and care [3].

How individuals suffering from depression perceive and interpret the incidents around them is also important. Perceived negative life events can lead to despair situations where signs of depression reveal. The level of depression may rise when individuals cannot perceive the social support [4]. For this reason, it is important for the individual with diabetic foot to perceive social support.

Social support is defined as the material and moral support provided to the individual under stress or in a difficult condition by the people around him/her [5]. Social support provides faith to the individual and leads him to cope with the stress-filled events more effectively.

Identifying the correlation of level of perceived social support of individuals suffering from diabetic foot with depression is important for the treatment process and to form appropriate solutions. Supportive plans are needed to be made for individuals with this level of diabetic foot and depression.

## Background

As a lifelong disease, diabetes impairs the quality of life by limiting the eating and drinking habits and by bringing out the risk of kidney, eye, cardiovascular and diabetic neurological diseases in the long run. Loss of health might result in mourning, grief, rebellion, denial, anxiety, rage and sometimes these feelings might overcome the patient’s coping skills leading to depression [1]. How individuals suffering from depression perceive and interpret the incidents around them is also important [4]. Accordingly, the determination of the correlation of the depression with the perceived social support level by the patients with diabetic foot was programmed and performed in order to take essential precautions, to generate proper solutions and treatment process and to make supportive plans for patients with developing diabetic foot and depression.

Basic objectives of this study are as follows: identifying the level of depression and perceived social support of individuals with diabetic foot, defining the influence of biophysiological, psychological, environmental, sociocultural and politico-economical factors on depression and perceived social support levels of individuals with diabetic foot.

## Method

### Study plan

Individuals at or over the age of 18 with diabetic foot that are in a suitable physical and cognitive state to answer the forms intended to be performed in the study who accepted to participate in the study and who applied—among the institutions within the Mersin municipality borders—to the Internal Sciences, Surgical Sciences services, Plastic Surgery Polyclinic Wound Healing Unit, Internal Diseases Endocrinology and Metabolism Diseases Polyclinic of Mersin University Health Research and Practice Center; Foot Health Unit, Endocrinology and Metabolism Diseases Polyclinic of Mersin State Hospital; Internal Diseases, Orthopedics and Dermatology services of Mersin Toros State Hospital between July 1^st^ 2011 and January 31^st^ 2012 were recruited to the study.

### Ethical aspects of research

Necessary permits for the conduct of the study were taken from Mersin University Clinical Research Ethics Committee (Reference number of the document:B.30.2.MEÜ.0.05.06.00125).

### Data collection

The data was collected by meeting face to face the individuals at or over the age of 18 with diabetic foot and by making use of the patient files after obtaining the official consents from the relevant institutions and written consents from the patients.

Study data was collected using the “Personal Information Form” which includes introductory information about individuals with diabetic foot, “Beck Depression Scale” which is applied to determine emotion status of individuals and “Multidimensional Scale of Perceived Social Support” which is applied to determine the level of social support individuals perceive.

Personal information form was generated as a result of relevant literature search in order to identify some socio-demographic, disease and treatment related characteristics that are thought to affect the degree of depression and the perceived social support of patients with diabetic foot.

#### Beck Depression Scale: (BDS)

In our country, the validity and reliability of the scale developed by Beck et al. in 1961, was studied by Hisli (1989) [6], respectively. The scale consists of 21 symptom categories and in each category there are 4 options. The highest score that can be obtained from the scale is 63. High total score indicates that degree and intensity of depression is high. In the scale; score of 0–10 indicates that there is no symptom of depression, score of 11–17 demonstrates mild depression, 18–29 moderate depression and 30–63 serious depression [6].

#### Multidimensional Scale of Perceived Social Support (MSPSS)

The validity and reliability of the scale that was developed by Zimet et al. in 1988, was studied in our country by Eker and Arkar in 1995. The scale consists of 12 items in total. MSPSS is a likert type scale that is organized in 7 stages as “Definitely no 1, 2, 3, 4, 5, 6, 7 definitely yes”. The scale has three subgroups that reflect the support sources such as family, friend, special person and each subgroup consists of 4 items. Among the items in the scale; 3, 4, 8, 11 evaluate family support; 6, 7, 9, 12 evaluate friend support and 1, 2, 5, 10 evaluate special person support. The minimum score that can be obtained from the subscales is 4 and the maximum score is 28. The minimum score that can be obtained from the total scale score, which is the sum of the subscale scores, is 12 and the maximum score is 84. A high score obtained from the scale indicates a high level of perceived social support [7].

#### Statistical analysis of data

Analyses were obtained from SPSS 11.5 (Statistical Package for The Social Sciences Windows) and MedCalc 11.6.1 packaged software. Tests of normality related to continuous measurements were obtained by Shapiro Wilk Test and were determined to show normal distribution. After Beck Depression scores were coded as moderate and serious depression, their comparison with socio-demographic characteristics was tested by Pearson chi-square, Fisher Exact and Likelihood Ratio chi-square tests. Number and percentage values were given as descriptive statistics. Student *t*-test and one-way analysis of variance test were used for the comparison of social support scores and sub-dimensions with socio-demographic characteristics. The control of the homogeneity of variances for variance analysis was done with Levene test. One-way ANOVA was used for the homogeneity of variance ensured parameters; Welch test was used when the homogeneity of variance was not ensured. For the pairwise comparison; Bonferonni test was used in the cases that the variances are homogenous and Games Howell test was used when the variances are not homogenous. Average and standard deviation values were given as the descriptive statistics. *p* < 0.05 was accepted as the statistically significant.

## Results

Among the individuals; 56.3 % are men, 81.3 % are between the ages 31 and 50, 37.5 % are primary school graduates, 60.9 % applied for the outpatient treatment, 65.6 % are married. Among them, 35.9 % are housewives, 36.7 % live with their parents. It was determined that income of 69.5 % of individuals are lower than their expenses, 41.4 % have moderate financial problems (Table 1).

**Table 1:** Distribution of individuals with diabetic foot with respect to their socio-demographic features

Variables	Number(n)	Percentage(%)
Sex		
Female	56	43.8
Male	72	56.3
Age group		
18–30	13	10.2
31–50	104	81.3
51 and over	11	8.6
Treatment type		
Inpatient	50	39.1
Outpatient	78	60.9
Marital status^a^		
Married	84	65.6
Single	44	34.4
Education status^b^		
Illiterate	37	26.6
Primary school	48	37.5
High school and over	46	35.9
Occupation		
Housewife	46	35.9
Worker	27	21.1
Official	10	7.8
Farmer	14	10.9
Retired	31	24.2
Level of income^c^		
Income more than expenditure	3	2.3
Income less than expenditure	89	69.5
Income equal to expenditure	36	28.1
People you live with^d^		
I live alone	28	21.9
With my family	39	30.5
With my mother/father	47	36.7
With someone out of family	14	10.9
Total	128	100

^a^The marital status of “single”, “widow” and “separated/divorced” are merged together as “single”

^b^The education status of “elementary school” and “secondary school” are merged together as “primary school”, the education status of “high school”, “university” and “postgraduate” are merged together as “high school and over”

^c^The level of income “no income” and “income less than expenditure” are merged together as “income less than expenditure”

^d^“I live with my partner” option in people you live with topic is merged with “I live with my family”

Among the individuals, 46.1 % are diabetic patients for 6–10 years, 39.8 % do not go for a control regularly, 50.8 % hospitalize due to diabetes 4–6 times a year. In terms of foot care, 79.7 % of individuals do not their feet regularly, 50.8 % make their foot care themselves, 51.6 % are not compatible with the diabetes treatment. It is determined that, 61.7 % of the individuals attended to an education about the disease and 47.7 % receive nutrition and insulin treatment (Table 2).

**Table 2:** Distribution of features regarding diabetes and diabetic foot

Variables	Number (n)	Percentage (%)
Duration of your diabetes		
0–5 years	2	18.8
6–10 years	4	46.1
11–15	59	16.4
16–20	21	4.7
21 years and over	6	14.1
Annual hospitalization frequency		
0-3 times	37	28.9
4–6 times	65	50.8
7 and over	26	20.3
Do you regularly maintain the controls of your diabetes?		
Yes	35	27.3
No	51	39.8
Partially	42	32.8
What the type of treatment against diabetes do you receive currently?		
Nutrition therapy + oral antidiabetic	41	32.0
Nutrition therapy + insulin	61	47.7
Nutrition therapy + oral antidiabetic + insulin	26	20.3
Do you comply in the treatment and care of diabetes?		
Yes	62	48.4
No	66	51.6
Can you perform your foot care personally?		
Yes	65	50.8
No	63	49.2
Do you check your feet often?		
Yes	26	20.3
No	102	79.7
Have you participated to a training regarding your problem yet?		
Yes	79	61.7
No	49	38.3
Total	128	100

In the performed statistical evaluation, a significant negative correlation was determined between the Beck Depression Scale mean scores and the mean of MSPSS family support, friend support, special person support sub-dimensions and scale total score (*p* < 0.01).

## Discussion

Besides basically being a chronic physical disease that is related to endocrine system, Diabetes Melitus (DM) is a case that might cause a series of mental, sentimental, social, psychosexual problems to the patient. By affecting brain functions it might cause psychiatric disorders; also in correlation with the detection and the effects of the disease to the living space of the patient, psychiatric charts might appear. These charts accompanying DM affect disease appearance, intensity, progress and response to the therapy. Holistic approach to the diabetic patient requires, in addition to physical therapy, diagnosis and therapy of organic, mental, sentimental, psychophysiologic and psychosocial charts that are accompanying the disease. In diabetic individuals, emotional reactions, adaptation difficulties and depressive disorders are the most commonly encountered mental disorders [6, 8].

It is also important how depressive individuals perceive and interpret the events happening around them. Negatively perceived life events might generate desperation that causes the depression symptoms to appear. The important point here is the correlation between the perception of negative life events and appearance of desperation. As a result, it is important for patients with diabetic foot to perceive the social support [4].

Social support is defined as the moral and material support provided by the relatives (partner, family, friends) to the individuals who are under stress or in a difficult situation [5]. Social support gains confidence to the individual and adds positive aspects to his/her life; also enables the individual to struggle more efficiently when a stressful situation is encountered [9].

Accordingly, the determination of the correlation of the depression with the perceived social support level by the patients with diabetic foot was programmed and performed in order to take essential precautions, to generate proper solutions and treatment process and to make supportive plans for patients with developing diabetic foot and depression.

Most of the patients (46.1 %) are suffering from diabetes for 6–10 years and approximately half of them (50.8 %) hospitalize due to diabetes 4–6 times a year (Table 2). In the study of Çıtıl et al. [10], most of the patients are suffering from diabetes for 1–5 years; in the study of Bahar et al [2], one third of the patients are suffering from diabetes for 1–5 years and the other one third of the patients are suffering from diabetes for 6–10 years. Also in the study of Bahar et al. [2], few of the patients hospitalize due to diabetes. Because hospitalization increases susceptibility to depression as a consequence of the change of the habitat; for the prevention of hospitalization it is suggestible that patients should go for a checkup frequently and patients should be informed and awareness should be raised for the implementation of regular treatment and care.

Most of the patients (39.8 %) do not have diabetes control regularly. Many of the patients (21.9 %) go for the control once a month or once 4–6 months (Table 2). According to the results of the study conducted by Çıtıl et al. [10], most of the patients go for physician control regularly. As the regular physician control is required to avoid disruption of the treatment of diabetic patients and to prevent complications; the high depression degree of the patients who were participated in the study and the fact that they do not go to control regularly, pose a risk for the disruption of the treatment and the development of complications.

Approximately half of the patients (48.4 %) stated themselves to be adapted to the diabetes treatment and care. Again half of the patients (50.8 %) are capable of doing their foot care themselves (Table 2). This finding is desired as patients being adapted to the treatment and doing foot care are required to prevent development of diabetic foot. Frequent check of the feet is required for the prevention of diabetic foot; still the statement of most of the patients (79.7 %) that they do not check their feet frequently appears to be an important problem (Table 2). This finding is compatible with the results of the study conducted by Harwell et al. [5]. Most of the individuals participated in the study (61.7 %) attended to an education about their disease (Table 2). In the study of Çıtıl et al. [10], approximately 3 out of 4 patients did not take an education about their disease. In the face of this result, it is seen that patients in the study group were given more education about the disease. Regular patient education about diabetes and its care and frequent follow up of the patients after the education are important requirements.

Individuals with chronic disease need support, acceptance, understanding and meaningful explanation in order to cope with the disease and the secondary problems brought about by the disease and to resolve their health problems. Long duration of disease was thought to increase the degree of depression; however, no significant correlation between the duration of disease and the degree of depression was observed in our study. This result might indicate that patients adapt to live with diabetes and diabetic foot and diabetes does not affect their lives dramatically. It might be said that as the disease duration prolongs, disease progresses and the diabetic complications develop, patients are under the risk of depressive disorder. In the study of Paschalides *et al*. [11], it was reported that the reasons of anxiety and depression in diabetic patients are negative thoughts of the patients about diabetes, more negative perceptions of the diabetic patients about the events, long duration of the disease and awareness of the complications caused by the disease. In our study, a significant correlation between the hospitalization frequency and the degree of depression was not found. However, in patients with the disease duration more than 5 years and hospitalization frequency more than 3, serious symptoms of depression were observed. Consequently; it might be said that as the disease duration prolongs, uncertainty and doubts decrease due to embracement of the disease; however dependency in performing activities of daily living increases; also depending on the effects of diabetes-related restrictions, losses or chronic fluctuations of blood glucose level on central nervous system, depression come into prominence.

Statistically significant correlation (*p* < 0.05) was determined between the maintenance of regular diabetes control and the degree of depression among the patients participated in the study. Moderate symptoms of depression for individuals having their diabetes control regularly or irregularly and serious symptoms of depression for individuals having their diabetes control partially, were determined (Table 3). This result is compatible with the study results of Miranda and Munoz [12] and Yücel et al. [13]. The degree of depression affects the regularity of diabetes control. It is thought that individuals who are provided to participate in treatment, believe in recovery and accept the disease, are more regardful about the controls and care; however, they need more support.

**Table 3:** Distribution of features regarding diabetes and diabetic foot according to BDS

Variables		Moderate depression		Severe depression		*x*2
		Number (n)	Percentage (%)	Number (n)	Percentage (%)	P
Duration of your diabetes	0–5 years	0	0	24	19.7	
	6–10 years	2	33.3	57	46.7	5,202
	11–15	2	33.3	19	15.6	0,267
	16–20	0	0	6	4.9	
	21 years and over	2	33.3	16	13.1	
Annual hospitalization frequency	0–3 times	2	33.3	35	28.7	
	4–6 times	4	66.7	61	50	2,823
	7 and over	0	0	26	21.3	0,244
Do you maintain the controls of your diabetes regularly?	Yes	4	66.7	31	25.4	
	No	2	33.3	49	40.2	6,685
	Partially	0	0	42	34.4	0,035
Do you check your feet often?	Yes	2	33.3	24	198.7	0,585
	No	4	66.7	98	80.3	0,601
Do you have any physical disorder?	Yes	2	33.3	2	1.6	7,551
	No	4	66.7	120	98.4	0,011
Do you think your disorder affects you psychology?	Yes	4	66.7	112	91.8	4,253
	No	2	33.3	10	8.2	0,039
Are you interested in your spare time activities and hobbies as much as you used to be before the disorder?	My interest is same as it used to be	0	0	8	6.6	
	It is a little less compared to before	0	0	38	31.4	
	It is significantly less compared to before	4	66.7	37	30.6	10,152
	Very less or no interest	2	33.3	38	31.4	0,017

Statistically significant difference (*p* < 0.05) was determined between the case that patients participated in the study have another physical disease and the case that this disease affect their mental state. Serious degree of depression was observed in the patients who do not have a physical disease besides diabetic foot (Table 3). In parallel with our study; as a result of the study of Glasgow *et al.* [14], it is stated that presence of another disease in addition to diabetes affects quality of life negatively and it is especially recognized in functional aspects and it increases the score of depression. In the study of Bursa [15], when the depression symptoms of handicapped individuals with another disease are examined; in parallel with our study, depression symptoms were determined in more than half of the handicapped individuals with another disease (61.3 %).

Serious degree of depression was observed in individuals who think that having diabetic foot affect mental state. According to the results of the study of Eren and Erdi [16] and Leedom et al. [8]; the degree of depression is high in DM patients who have complications; on the other hand, there is no significant correlation between the depression and the complications according to the results of the study of Bahar et al. [2] and Gülseren et al [17]. Diabetic complications are problems that decrease the quality of life and bring important restrictions to the life of the diabetic patient. Diabetic patients often hospitalize due to poor glycemic control and complications. Lack of information about the disease future concern make individuals go through psychological problems [2].

Total social support score, family support score and special person support score of the individuals in the groups of 0–5 or 6–10 years of duration of diabetes, are higher than the scores of the individuals in the groups of 11–15, 16–20 and 21 and over 21 years (Table 4). In the study of Dayapoğlu and Tan [18], that is performed on stroke patients, the mean perceived social support score was determined to be highest between 3^rd^ and 9^th^ months of the disease. As the duration of the disease and care prolong, the individuals who undertake the care remain under intense stress as a consequence of giving constant care and this emotionally exhausted individuals become distanced to the patient.

**Table 4:** Mean MSPSS scores according to diabetes and diabetic foot related characteristics of individuals

Variables		Social support Avg + SD	Family support Avg + SD	Friend support Avg + SD	Special person support Avg + SD
Duration of diabetes	0–5	41,5 ± 11,4	15,2 ± 8,3	9,3 ± 4,7	17 ± 8,3
	6–10	41,5 ± 15,9	15,7 ± 7,2	8,8 ± 4,8	17 ± 8,5
	11–15	34,6 ± 21,4	14,4 ± 8,8	11,3 ± 6	12,9 ± 10
	16–20	30,7 ± 19,2	6,0 ± 2,3^a,b^	8 ± 4,4	9,3 ± 8,3^b^
	21 and over	28 ± 18,7^a,b^	10,1 ± 7,2	9,1 ± 6,1	8,9 ± 7,2^a,b^
F & p		3,019 &0,020	14,930& < 0,001	1,023 & 0,398	4,392 & 0,002
Annual Hospitalization Frequency	0–3 times	42,5 ± 14,1	15,5 ± 7,9	9,5 ± 4,3	17,5 ± 8,5
	4–6 times	40 ± 17,9	14,4 ± 8,1	9,5 ± 6	16,2 ± 8,7
	7 and over	26,5 ± 15,3^c,d^	8,5 ± 5,9^c,d^	8,7 ± 4,3	7,7 ± 6,6^c,d^
F & p		6,256 &0,001	10,023& <0,001	0,367& 0,694	17,186& <0,001
Regular Control of Diabetes	Yes	45,4 ± 17,9	16,6 ± 8,8	10,9 ± 5,2	17,9 ± 9,2
	No	32 ± 15,1^e^	11,2 ± 6,9^e^	8,7 ± 4,7	11,2 ± 7,8^e^
	Partially	39 ± 16,9^f^	13,6 ± 7,9	8,8 ± 5,6	16,6 ± 8,8^f^
F & p F & p		7,012 & 0,001	5,005 &0,008	2,158 & 0,120	7,820 & 0,001
Do you think this disease affect your mental state?	Yes	36,4 ± 16,6	13,1 ± 7,9	9 ± 5,2	14 ± 8,8
	No	52,8 ± 17,5	17,5 ± 8,2	12,7 ± 4,4	22,7 ± 6,9
t & p		−3,244 & 0,002	−1,837 & 0,069	−2,390 & 0,018	−3,288 & 0,001
Did you experience psychological problems after the development of diabetic foot wound?	Yes	36,5 ± 16,5	13,1 ± 8	8,8 ± 5,1	14,1 ± 9
	No	45,3 ± 19,4	15,4 ± 8,1	11,6 ± 5	18,3 ± 8,6
t & p		−2,223 & 0,028	−1,206 & 0,230	−2,341 & 0,021	−2,025 & 0,045
Are you compatible with the treatment and care of diabetes?	Yes	40,1 ± 17,6	13,5 ± 8,3	9,5 ± 5,5	17 ± 9
	No	36 ± 16,8	13,5 ± 7,8	9,1 ± 4,9	12,7 ± 8,6
t & p		1,360 & 0,176 0,	033 & 0,974	0,480 & 0,632	2,782 & 0,006
At present How well is Your physical Performance To perform your Profession/ Labor/ Housework?	III-				
	conditioned	31,6 ± 17,1	10,7 ± 6,1	8,6 ± 5,4	11,2 ± 7,4
	Not so good	38,5 ± 15,3	14,3 ± 8,4^g^	8,7 ± 4,9	15,6 ± 9,1^g^
	Sufficient	45,3 ± 19,6^g^	15,5 ± 8,5 s	11,9 ± 5,1^g,h^	17,9 ± 9,5^g^
F & p		4,268 & 0,019	4,369 & 0,017	4,325 & 0,020	5,042 & 0,005

^a^The duration of diabetes “0–5 years ” statistically significant difference from the group

^b^Diabetes duration “6–10 years ” statistically significant difference from the group

^c^Frequency of hospitalization “0–3 times ” statistically significant difference from the group

^d^The incidence of hospitalization “ 4–6 times ” statistically significant difference from the group

^e^Regular control of diabetes “ yes ” no statistically significant difference from the group

^f^Regular control of diabetes “ no,” no statistically significant difference from the group

^g^Current occupation/work/home to do their jobs in terms of physical performance, “worst case ” statistically significant difference from the group

^h^Current occupation/work/home to do their jobs in terms of physical performance in terms of “not so good” uses the phrase statistically significant difference from the group

## Conclusions and suggestions

In the performed statistical evaluation, mean scores of Beck Depression scale and MSPSS family support, friend support, special person support sub-dimension and scale total scores were found to be in negative statistical correlation (*p* < 0.01) (Tables 5 and 6). This result demonstrates that as the family, friend and special person support of the individuals increase, the degree of depression decreases. This result is in parallel with the study of Kara and Mirici (2004) and Korkmaz and Tel (2010) [19, 20]. Social support positively affects physical and mental health by satisfying the individuals’ needs of love, compassion and self-respect. The case of not getting the needed help, causes the individuals to feel useless, worthless, desperate and causes depression.

**Table 5:** The distribution of BDS and MSPSS scores of the individuals

Scales		Potential distribution	Min._Max.	Avg. ± Sd
BDS		0–63	29–84	48,3906 ± 12,58623
	Family Sup	4–28	4–28	13,4922 ± 8,01376
	Friend Sup.	4–28	4–24	9,3203 ± 5,18923
	Special Person Sup.	4–28	4–28	14,8203 ± 9,01218
	Social Sup	12–84	12–77	37,9766 ± 17,28067
Total		24–231	53–241	124 ± 52,08207

**Table 6:** The correlation of MSPSS total and sub-dimension scores with BDS scores

	Family support	Friend support	Special person support
*r*	0,847^a^	0,533^a^	0,790^a^
Social Support			
*p*	<0,001	<0,001	<0,001
*r*	1	0,353^a^	0,558^a^
Family Support			
*p*	−	<0,001	<0,001
*r*	0,353^a^	1	0,113
Friend Support			
*p*	<0,001	−	0,205
*r*	0,558^a^	0,113^b^	1
Special Person Support			
*p*	<0,001	0,205	−
*r*	−0,498^a^	−0,411^a^	−0,279^a^
Beck Depression			
*p*	<0,001	<0,001	<0,001

^a^significant at the level of *p* < 0,01, ^b^significant at the level of *p* < 0,05

According to the results obtained from our study:

- Education of the patients should be grounded on learning models that consider cognitive-social factors.
- The concept of social support should be included in nursing education, in more detail.
- In the treatment and care of the patients with diabetic foot; anxiety and depression status of the patients, as well as physical status, should also be evaluated routinely. The individuals carrying the risk of depression and anxiety should be determined at early stage and provided to take professional care.
- The patient and the family should be guided to get familiar with social support sources and to use these sources effectively.
- Because the attitude of the patients affects the treatment and care in diabetes, diabetic patients should be given an education that is planned with respect to self-management education and afterwards their attitudes should be evaluated again.
- Medical staff should be ensured to be sensitive about the mental problems accompanying the physical disease.
- High support of the family to the individuals, who are dependent on others for the daily activities, is important to eliminate the feeling of being useless; so it is suggested that centers that families can receive consultancy from time to time are created.
